# Supplementary material for: TOMM40 regulates hepatocellular and plasma lipid metabolism via an LXR-dependent pathway
Source: Mol Metab. 2024 Nov 1;90:102056. doi: 10.1016/j.molmet.2024.102056 (PMC11600064; doi:10.1016/j.molmet.2024.102056)
Supplement: Multimedia component 1 [file mmc1.docx]

**SUPPLEMENTAL FIGURES:**


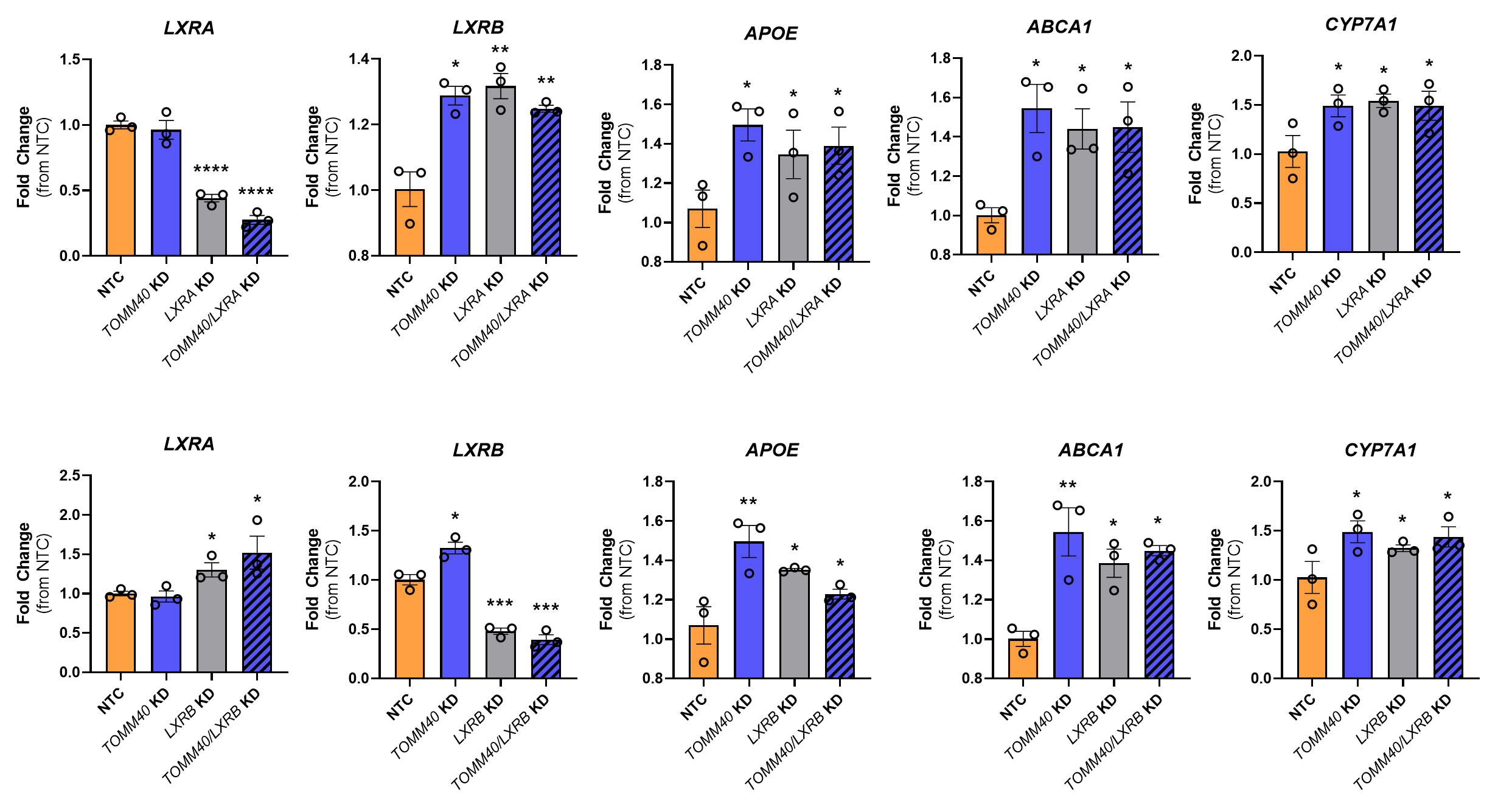


**Figure S1. LXRA and LXRB isoforms reciprocally maintain LXR expression and regulate downstream gene targets.** Relative mRNA transcript levels *of LXRA, LXRB, APOE, ABCA1,* and *CYP7A1*, compared between NTC vs. *TOMM40, LXRA, LXRB siRNA*s, singly or in combination, in HepG2 cells. *p<0.05, **p<0.01, ***p<0.005, ****p<0.001 vs. NTC by one-way ANOVA, with post-hoc Student’s t-test. (*n=3* biological replicates)


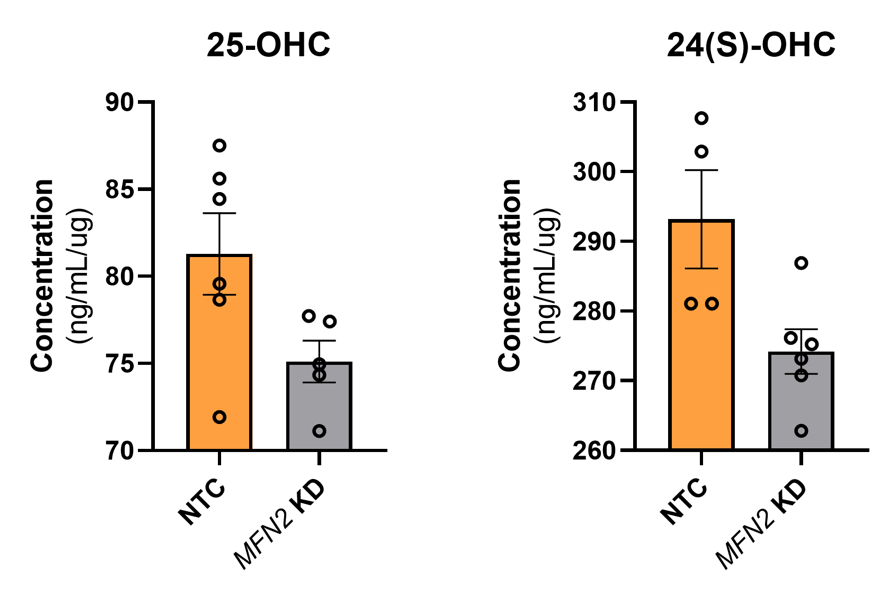


**Figure S2. *MFN2* KD does not affect enzymatic-derived oxysterols in HepG2 cells.** Analysis of enzymatic-derived 25-OHC levels and 24(S)-OHC in *TOMM40* KD vs. NTC HepG2 cells by ELISAs. (*n=3* biological replicates)


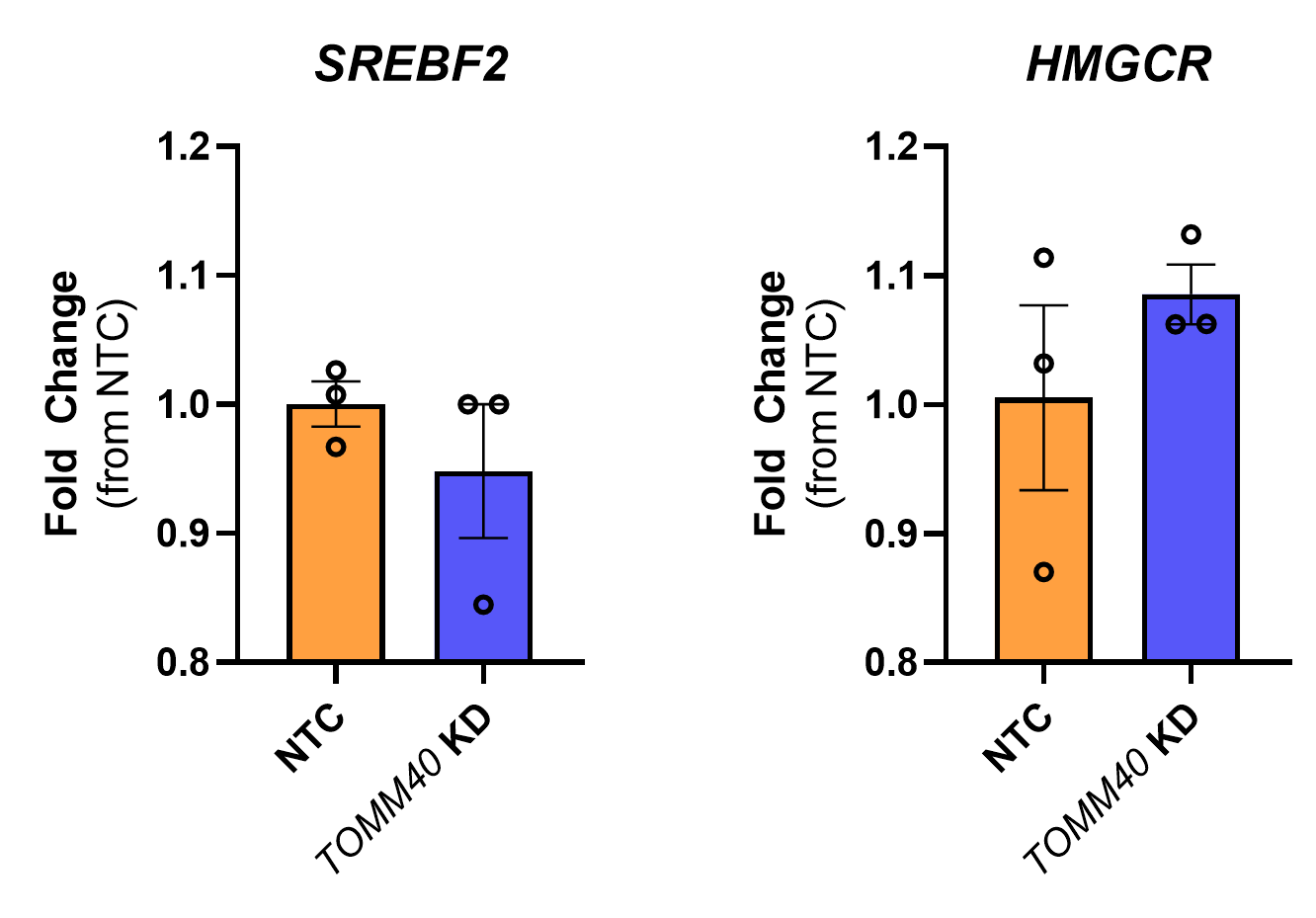


**Figure S3. *TOMM40* KD does not affects *SREBF2* or *HMGCR* mRNA transcript levels in HepG2 cells.** (*n=3* biological replicates)


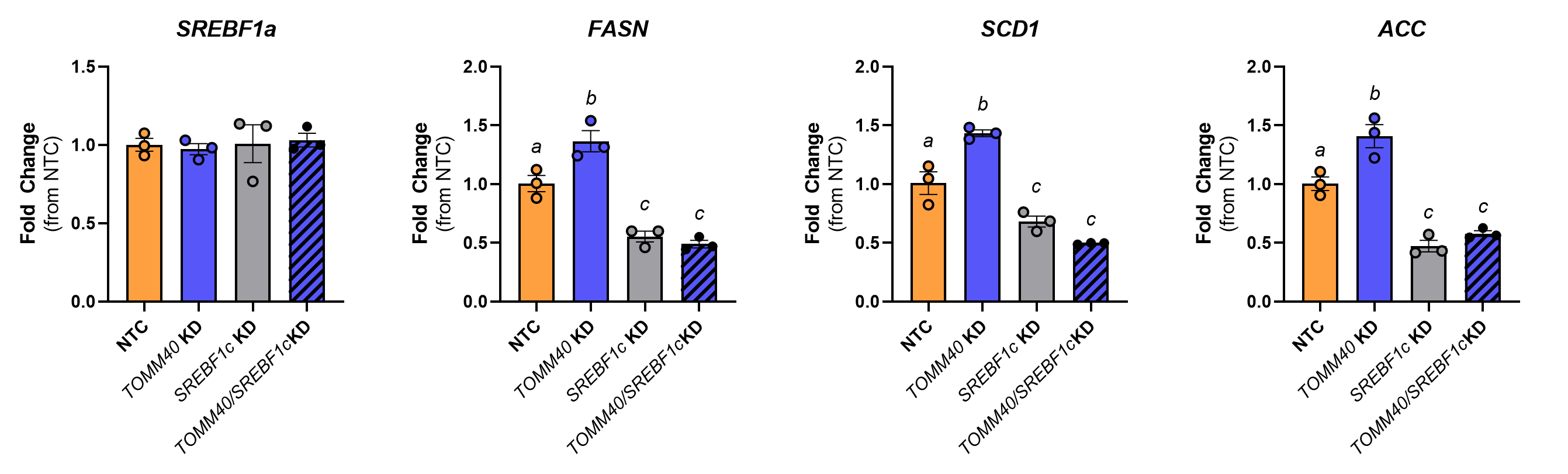


**Figure S4. *TOMM40* KD upregulates *FASN, SCD1*, and *ACC* mRNA transcripts via SREBF1c in *de novo lipogenesis* in HepG2 cells.** mRNA transcripts of *SREBF1a, FASN, SCD1,* and *ACC,* from NTC vs. TOMM40 KD HepG2 cells with or without *SREBF1c* KD were quantified by qPCR. p<0.05 for *a* vs. *b* vs. *c* vs. *d* by two-way ANOVA, with Sidak’s multiple comparisons test. (n=3 biological replicates)


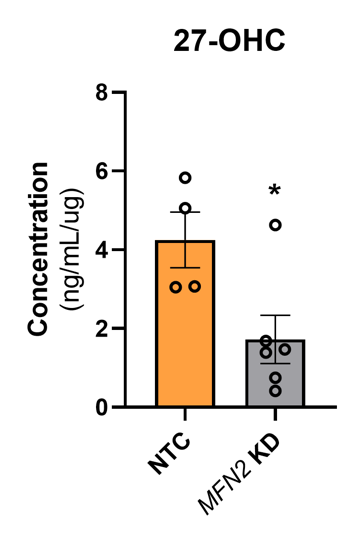


**Figure S5. MFN2 KD reduces 27-OHC levels in HepG2 cells.** Analysis of enzymatic-derived 27-OHC levels in NTC vs. *TOMM40* KD HepG2 cells by ELISA. *p<0.05 vs. NTC by one-way ANOVA, with post-hoc Student’s t-test. (n=3 biological replicates)


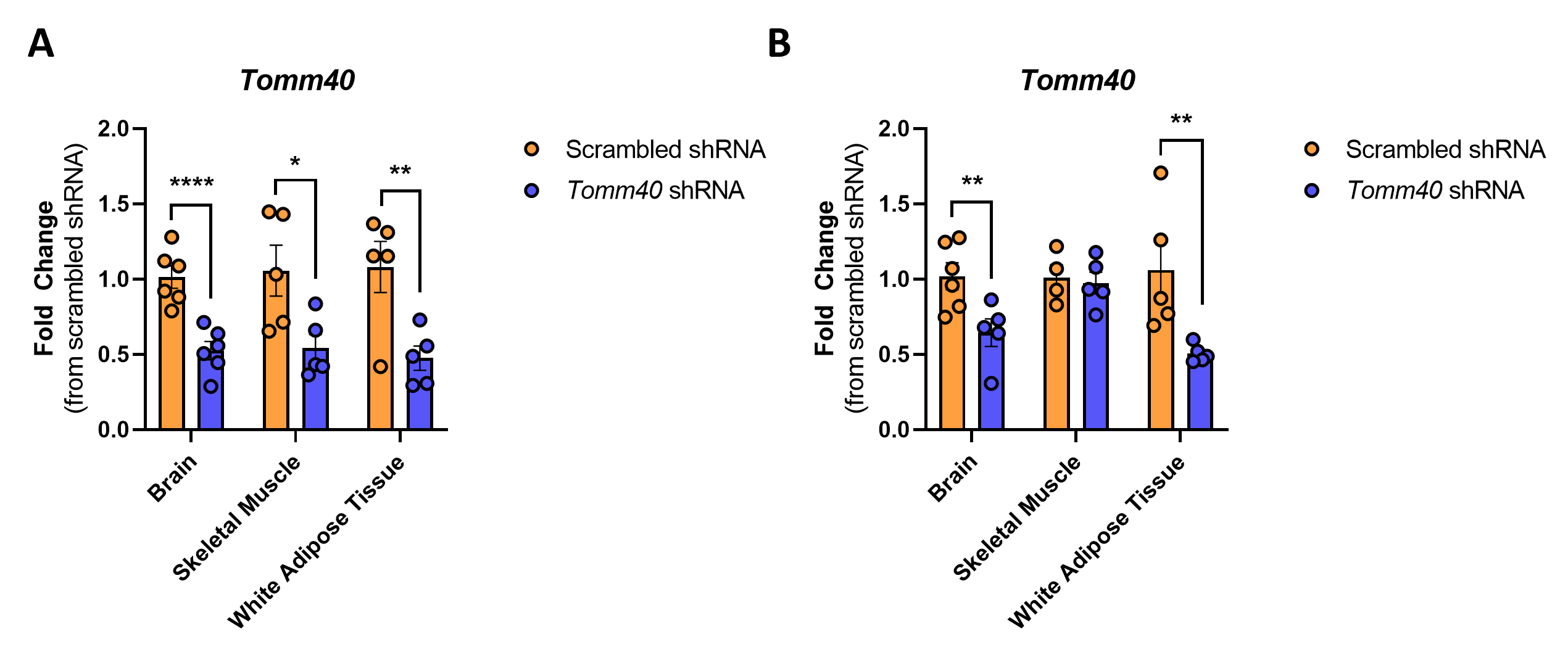


**Figure S6. *Tomm40* KD in gastrocnemius skeletal muscle, white adipose, and brain of AAV8-*Tomm40* shRNA C57BL/6J mice.** (A-B) mRNA transcripts of *Tomm40* from scrambled vs. *Tomm40* shRNA mice were quantified by qPCR in male (A) and female (B). *p<0.05, **p<0.01, ***p<0.005, ****p<0.001 vs. NTC by one-way ANOVA, with post-hoc Student’s t-test. (*n=5-6* mice/sex/group)


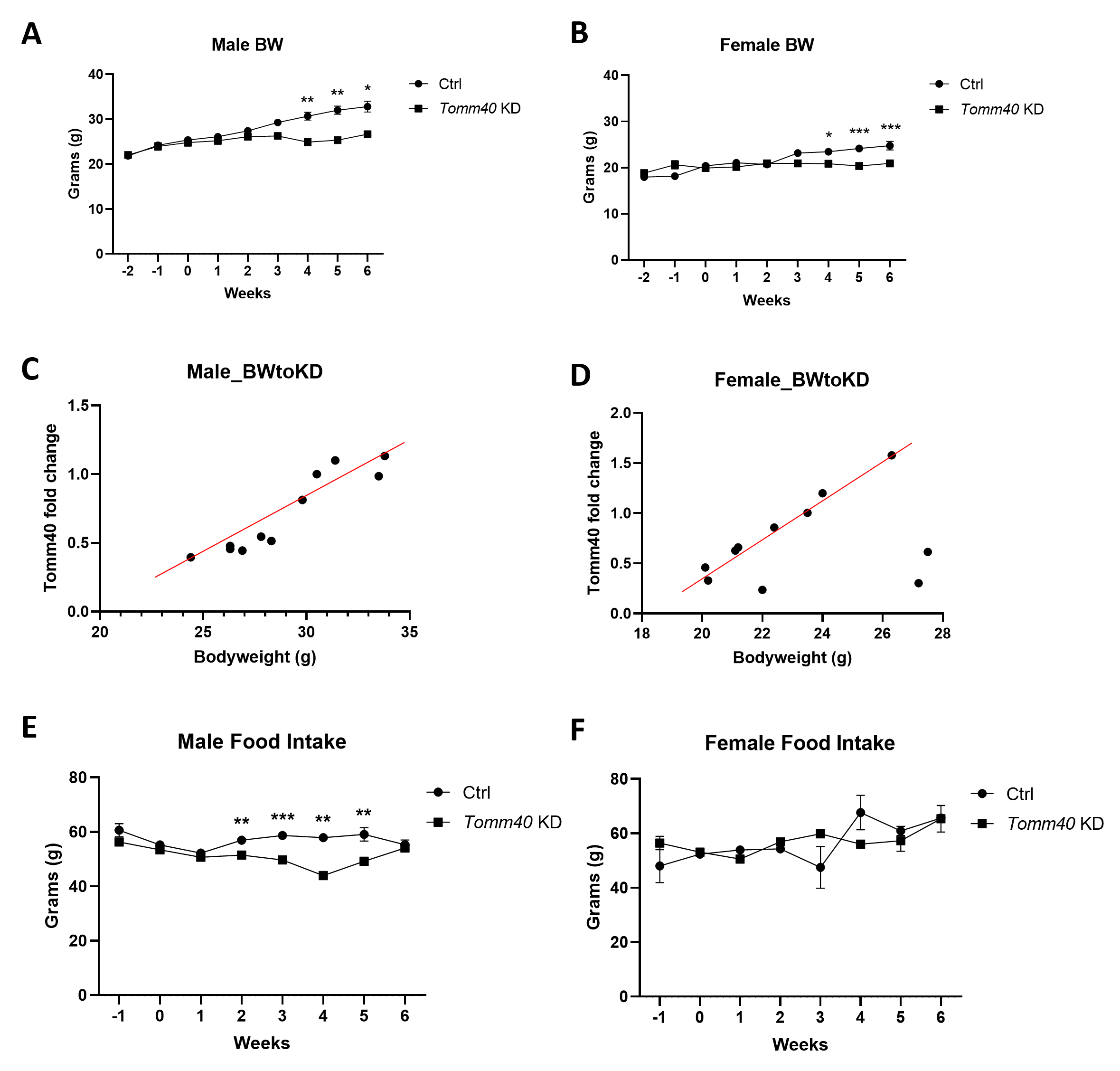


**Figure S7. Body weight and food intake measurements of AAV8-*Tomm40* shRNA C57BL/6J mice.** (A-B) Weekly bodyweight of male (A) and female (B) scrambled vs. *Tomm40* shRNA mice. (C-D) Comparison between *Tomm40* KD: bodyweight ratio in male (C) and female (D) mice. (E-F) Weekly food intake of male (E) and female (F) mice. *p<0.05, **p<0.01, ***p<0.005, ****p<0.001 vs. NTC by one-way ANOVA, with post-hoc Student’s t-test. (*n=5-6* mice/sex/group)


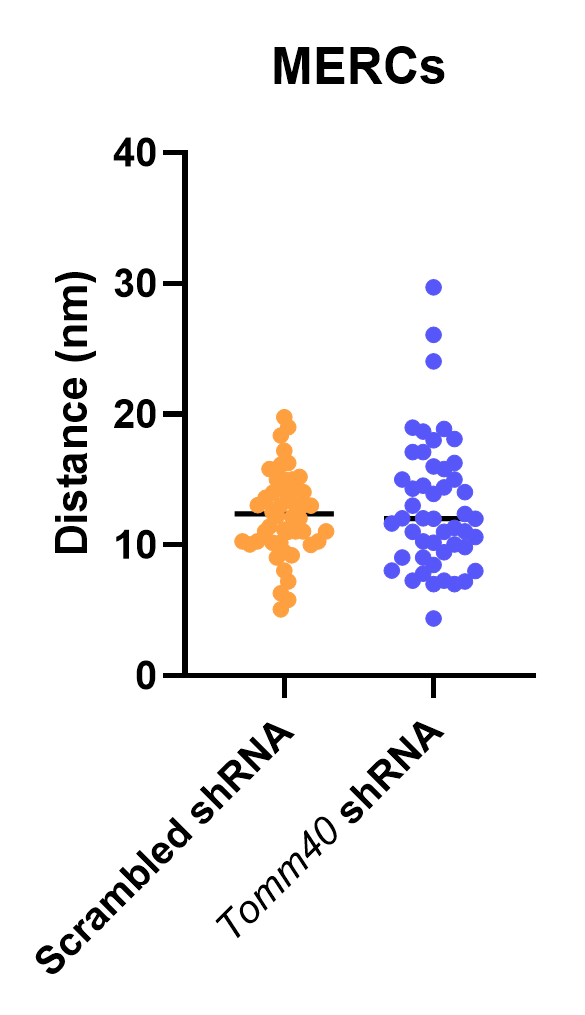


**Figure S8. Analysis of distance (nm) between MERCs in AAV8-*Tomm40* shRNA C57BL/6J female mice liver.** TEM images were analyzed by ImageJ software. (*n= 24-48* fields)


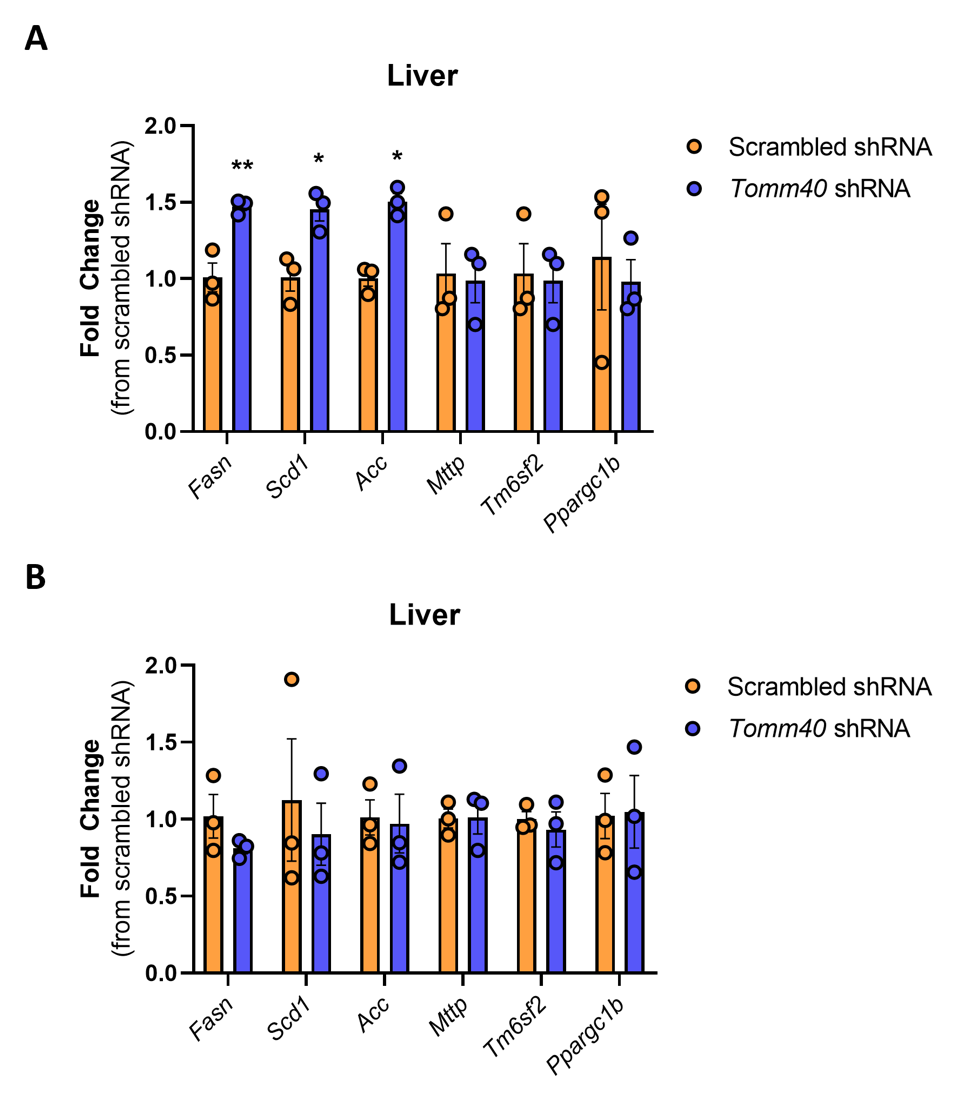


**Figure S9.** ***Tomm40* KD upregulates genes involved in *de novo lipogenesis* in AAV8-*Tomm40* shRNA C57BL/6J male mice liver.** mRNA transcripts from scrambled vs. *Tomm40* shRNA mice were quantified by qPCR in male (A) and female (B) mice liver. *p<0.05, **p<0.01, ***p<0.005, ****p<0.001 vs. NTC by one-way ANOVA, with post-hoc Student’s t-test. (*n=3* mice/sex/group)


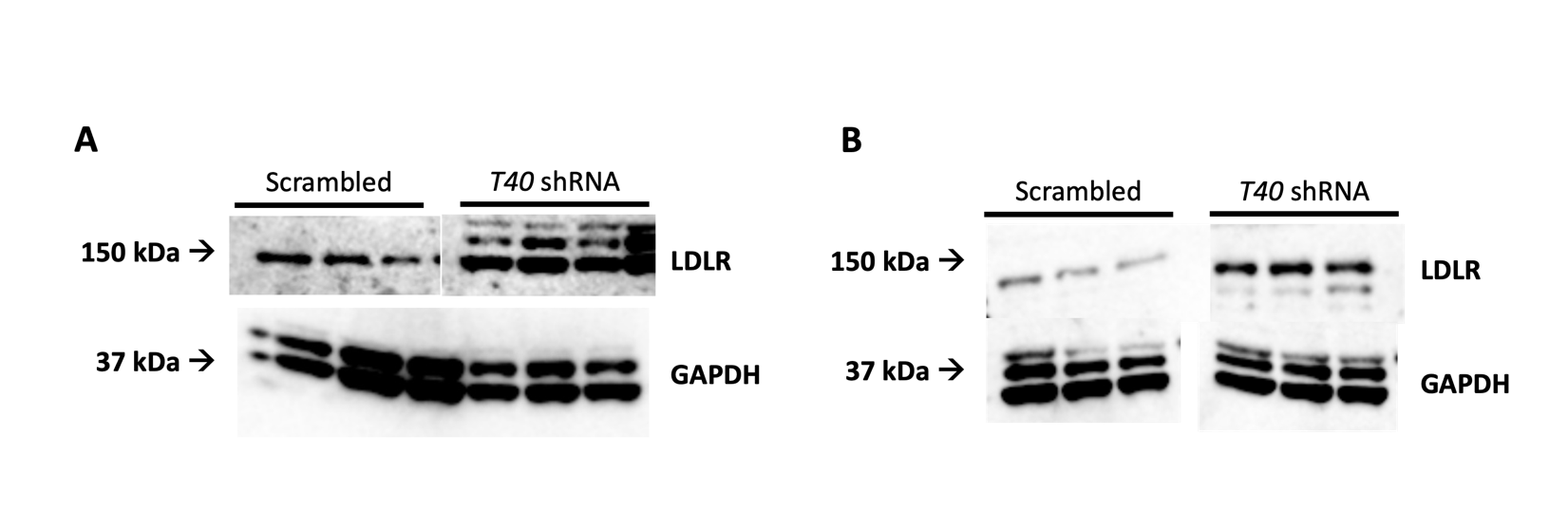


**Figures S10. Representative western blot of LDLR protein expression of scrambled vs. *Tomm40* shRNA in male (A) and female (B) mice liver.** GAPDH was used as the control.


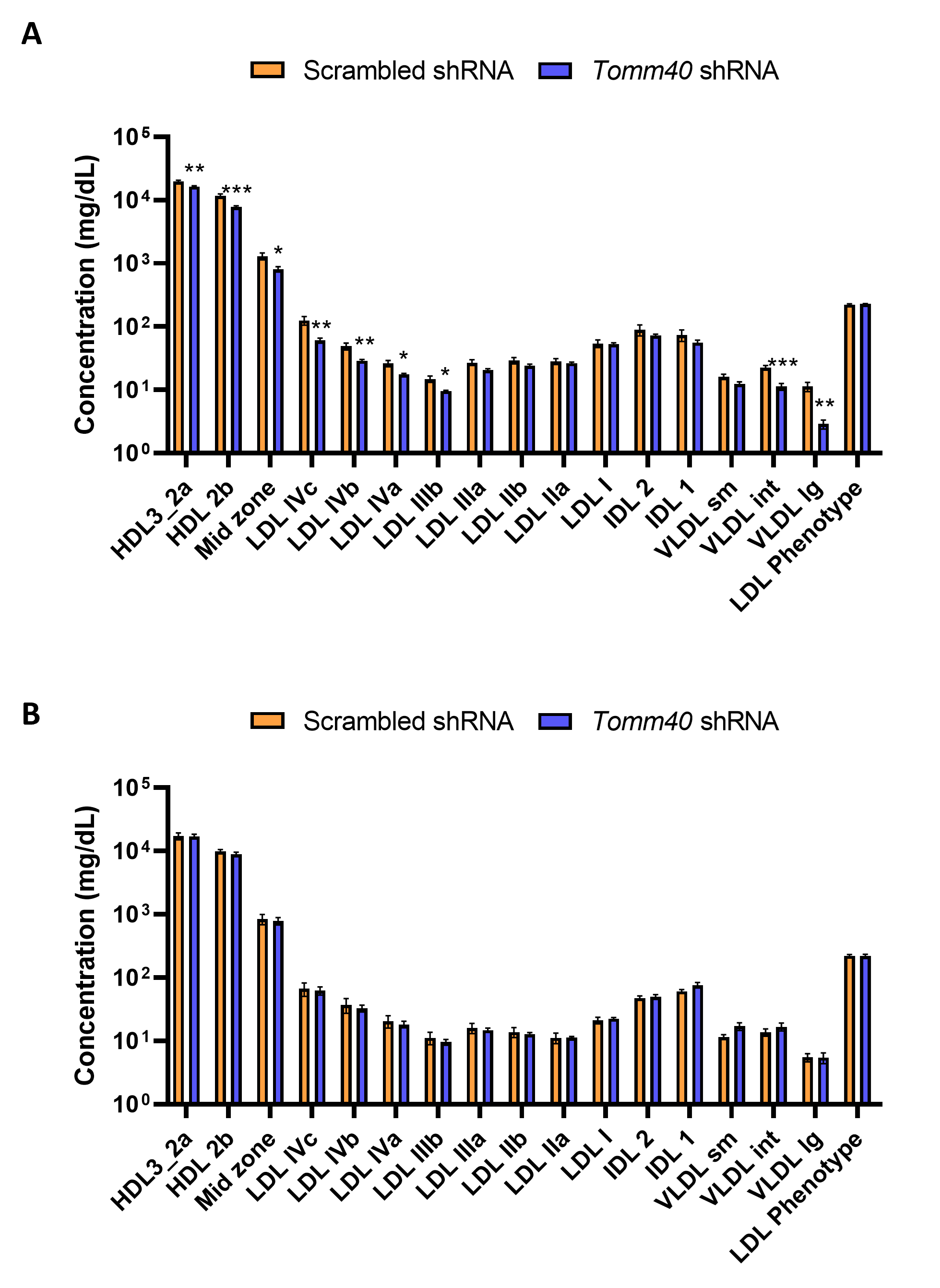


**Figure S11. Measurements of lipoprotein particle concentrations on mouse plasma.** Lipoprotein concentrations were quantified by ion mobility in (A) male and (B) female mouse plasma in *Tomm40* KD vs. scrambled control. Mouse plasma lipoprotein subfractions were analyzed based on human clinical lipoprotein particle classifications. *p<0.05, **p<0.01, ***p<0.005, ****p<0.001 vs. NTC by one-way ANOVA, with post-hoc Student’s t-test. (n=6 mice/sex/group)


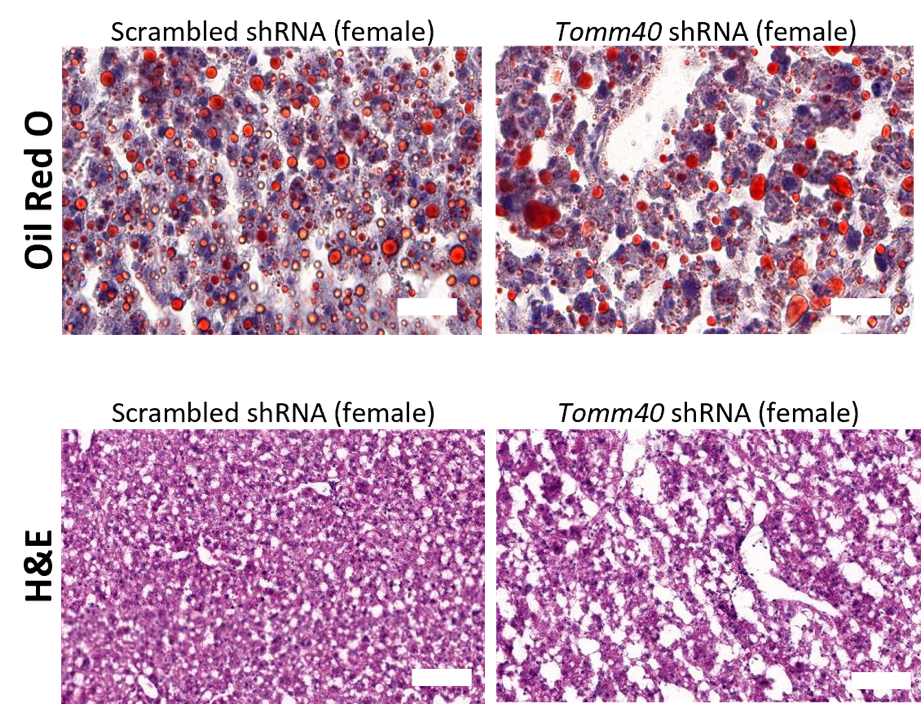


**Figure S12. Representative Oil Red O and Hematoxylin-Eosin stained liver samples of female mice.** (400 x magnification) Scale bars, 50 µm.


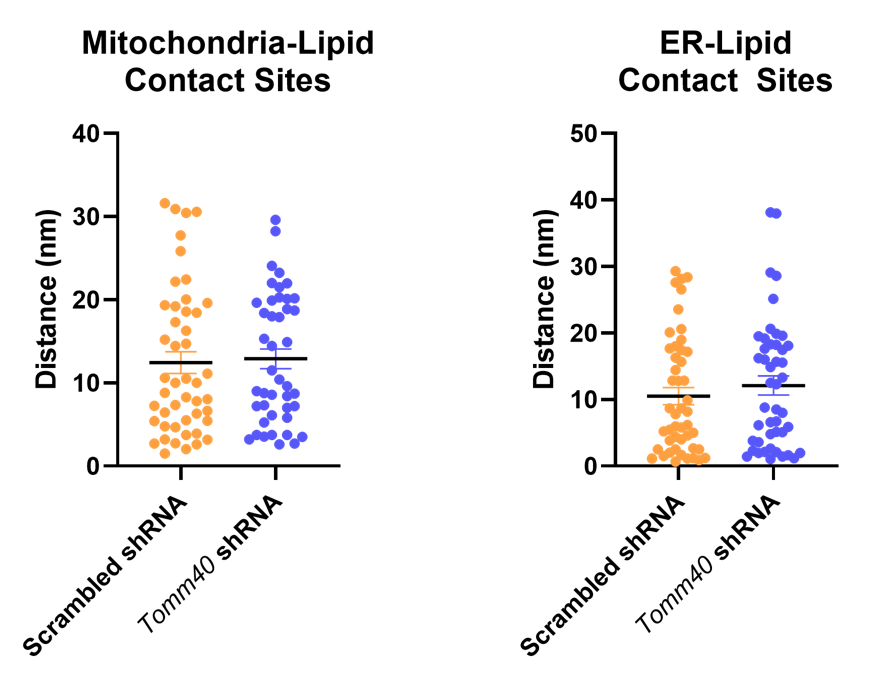


**Figure S13. Analysis of TEM micrographs indicating no differences in lipid droplet-ER and lipid-droplet mitochondria contact sites in female mice.** (A) Distance between mitochondria and lipid contact sites (nm), (B) Distance between ER and lipid contact sites (nm). (*n=24-48* fields)

**
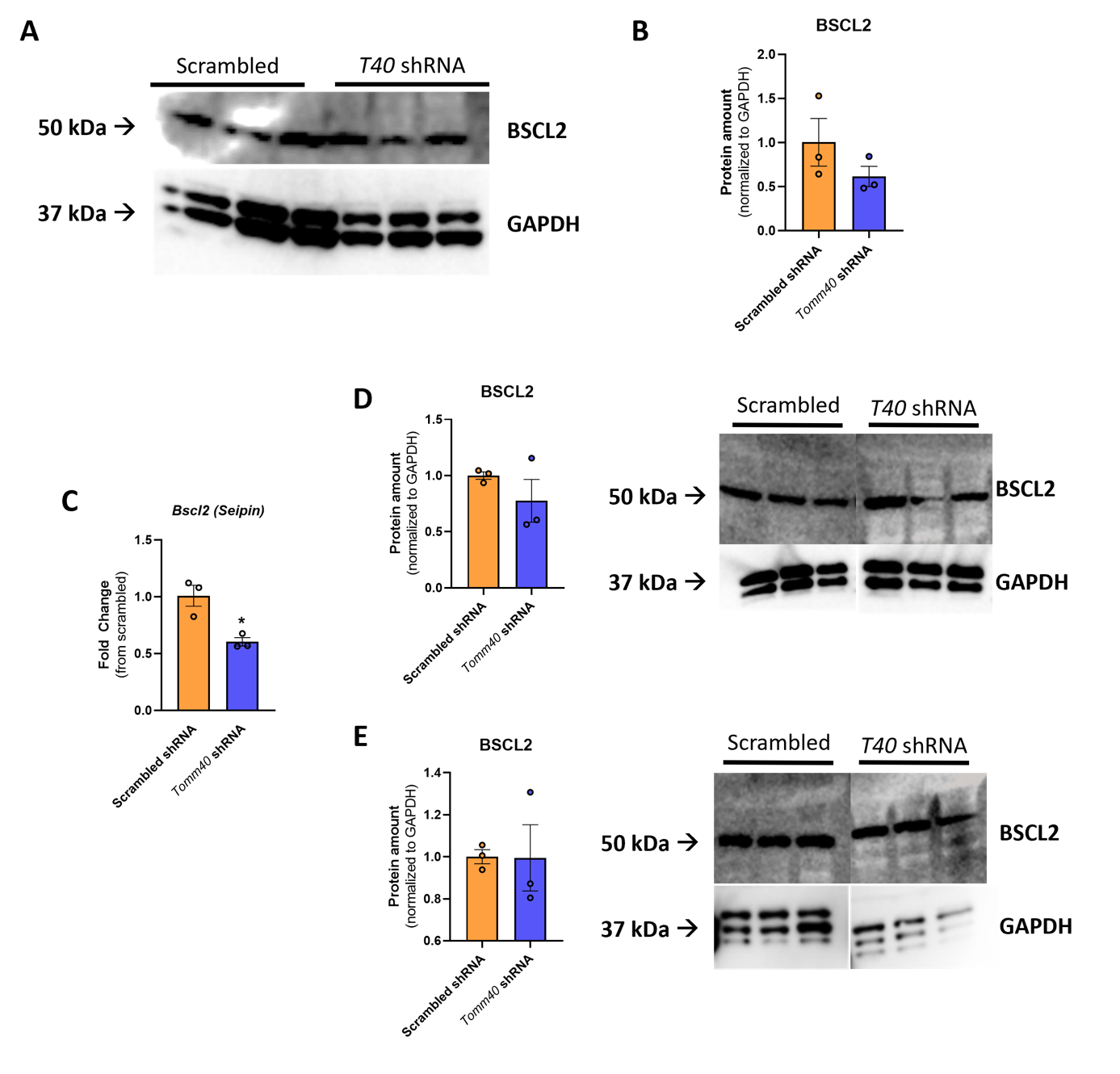
**

**Figure S14. Quantification of *Bscl2*/BSCL2 expression in mouse hepatic tissues.** (A) Representative western blot and (B) relative protein amount of BSCL2 protein expression in cytosolic fractions of scrambled vs. *Tomm40* shRNA male mice liver. (C) mRNA transcript levels of Bscl2 in scrambled vs. *Tomm40* shRNA female mice liver. Relative protein amount and representative western blot of BSCL2 protein expression in (D) mitochondria-associated membranes (MAMs) and (E) cytosolic fractions in scrambled vs. *Tomm40* shRNA female mice livers (GAPDH was used as the control). For all: *n=3* mice per group; *p<0.05, vs. scrambled shRNA by one-way ANOVA, with post-hoc Student’s t-test to identify differences between groups. Data are represented as mean ± SEM.


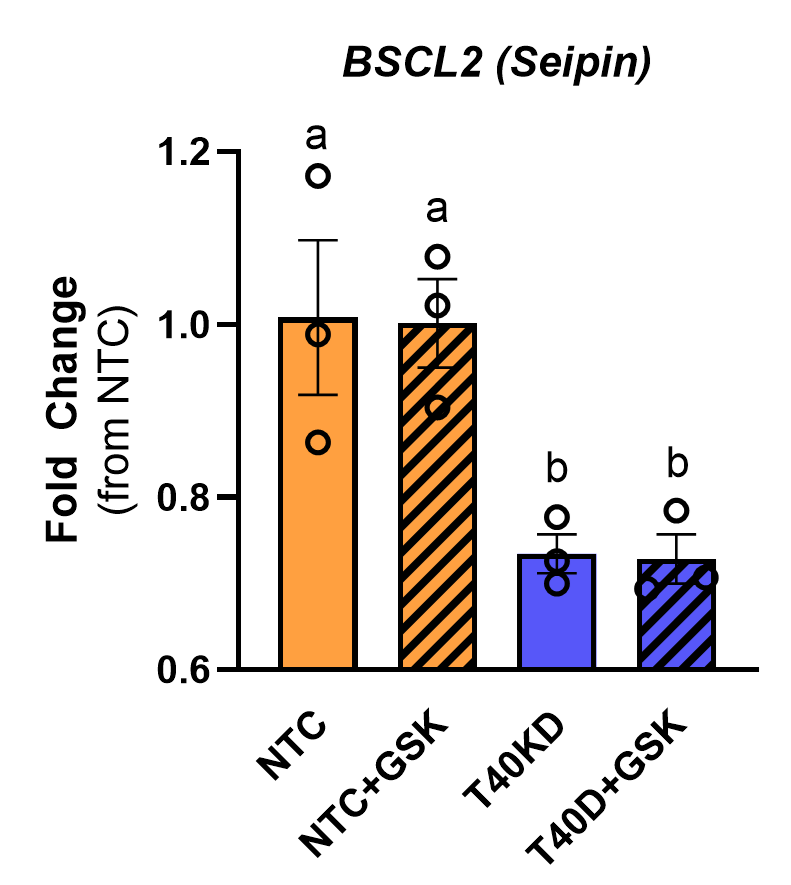


**Figure S15. mRNA transcript levels of *BSCL2* in HepG2 cells.** mRNA transcripts of *BSCL2* from NTC vs. TOMM40 KD HepG2 cells with or without addition of GSK2033 were quantified by qPCR. p<0.05 for *a* vs. *b* vs. *c* vs. *d* by two-way ANOVA, with Sidak’s multiple comparisons test. (n=3 biological replicates)

**
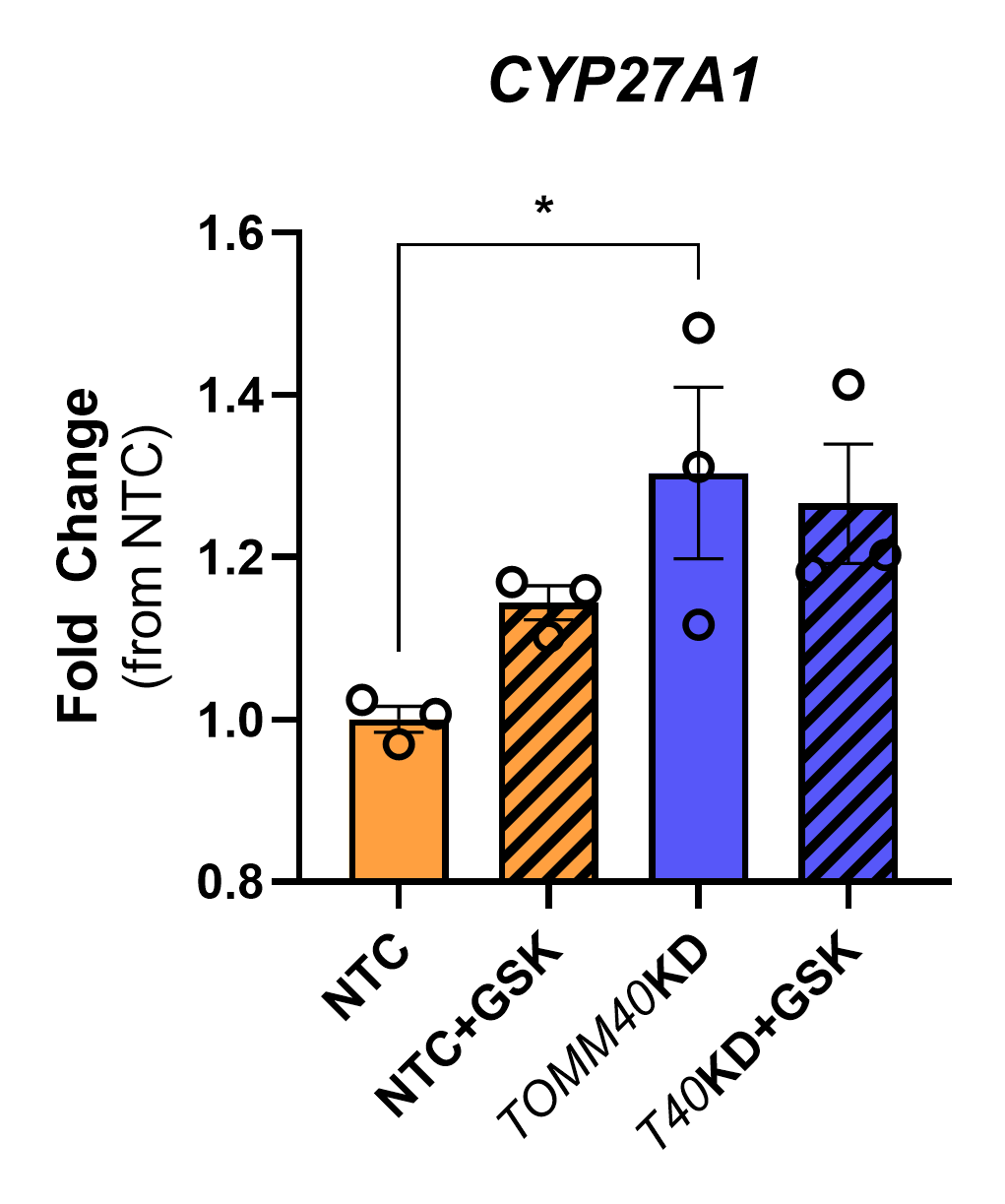
**

**Figure S16. CYP27A1 mRNA transcript levels.** mRNA transcripts of *CYP27A1* from NTC vs. TOMM40 KD HepG2 cells with or without addition of GSK2033 were quantified by qPCR. *p<0.05 vs. NTC by one-way ANOVA, with post-hoc Student’s t-test. (n=3 biological replicates)
